# Recommendations on Ultrasound‐Guided Hyaluronic Acid Soft Tissue Augmentation of the Upper Face in Asians

**DOI:** 10.1111/jocd.16759

**Published:** 2024-12-30

**Authors:** Jae Yoon Jung, Hosung Choi, Sang Chul Han, Rungsima Wanitphakdeedecha, Qian Luo, Lijun Zhou, Jung Im No, Woonji E. Jang, Hee‐Jin Kim

**Affiliations:** ^1^ Department of Dermatology Oaro Dermatology Clinic Seoul Republic of Korea; ^2^ Facial Plastic Surgery Department PIENA Aesthetic Medical Clinic in Gangnam Seoul Republic of Korea; ^3^ Department of Plastic Surgery HIT Plastic Surgery Seoul Republic of Korea; ^4^ Department of Dermatology Faculty of Medicine Siriraj Hospital, Mahidol University Bangkok Thailand; ^5^ Department of Aesthetic Surgery Beijing Phiskin Aesthetic Medical Clinic Beijing China; ^6^ Department of Plastic Surgery Chengdu Badachu Medical Beauty Hospital Chengdu Sichuan China; ^7^ Aesthetic Medical Team, LG Chem Ltd. Seoul Republic of Korea; ^8^ Department of Oral Biology Yonsei University College of Dentistry Seoul Republic of Korea

**Keywords:** forehead, hyaluronic acid filler, injection, temple, ultrasonography, upper eyelid

## Abstract

**Background:**

Ultrasonography allows real‐time imaging of facial soft tissue during hyaluronic acid (HA) filler injections. However, there is currently limited guidance relating to ultrasound‐guided HA filler placement in the upper face.

**Aims:**

To develop guidance for the effective use of ultrasonography to improve the safety of HA filler injection procedures.

**Methods:**

Through a series of online video conferences and anatomical studies conducted on a model and a cadaveric specimen, specialists from Korea, Thailand, and China drew recommendations for ultrasound‐guided filler injections of the upper face.

**Results:**

The authors outlined critical anatomical landmarks for safe HA filler placement to correct volume deficits or treat hollowness of the forehead, temple, and upper eyelids. They provide insights on treatment indications and injection techniques using ultrasound for safe HA filler placement. The recommendations are presented in this paper.

**Conclusions:**

Where there are anatomic variations in the vasculature of the forehead, temple, and periorbital area, ultrasound‐guided filler placement can provide an additional safety measure. Current evidence may give rise to standard practices incorporating ultrasound in injection protocols for the preventive and therapeutic management of adverse events.

## 1 Introduction

Hyaluronic acid (HA) fillers have become a popular choice for facial sculpting and rejuvenation due to their safety and tolerability profiles [1]. Nonetheless, adverse events following HA filler administration may occur [2]. While most of them are mild and transient (e.g., erythema, edema, bruising) [3], rare and more serious complications have also been documented (i.e., compromise of large‐caliber vessels leading to skin necrosis, hair loss, and embolisms such as stroke and vision loss from retinal artery occlusion) [3, 4, 5]. Achieving optimal patient outcomes requires expertise in anatomy, morphology, and product use; however, these factors alone do not guarantee a lack of complications [6]. Interindividual anatomical variations in the depth and distribution of facial vasculature exist, and these features are of particular importance in the upper face, where complex vascular patterns have been identified. In cadaveric specimens, the distribution pattern of the deep branch of the supratrochlear artery may be present in 55% of cases, as it supplies the deep aspect of the frontalis, or absent (45%), and only the deep branch of the supraorbital artery supplies the frontalis [7]. Consequently, guidelines for injecting fillers recommend precautionary measures such as palpation of arterial vessels, syringe aspiration before injection, use of blunt cannulas in more superficial layers, and retrograde, slow, steady, and low‐pressure injection [5, 8].

Ultrasonographic evaluation of the face allows for vascular mapping in aesthetics [9]. Studies on ultrasound‐guided filler placement suggest that it enables injectors to safely introduce HA fillers in various facial regions (i.e., temple, upper eyelids, cheeks) [10, 11, 12]. Ultrasonography may help to prevent severe or permanent complications associated with vascular compromise [13].

In this publication, specialists in aesthetic medicine/anatomy and dermatology from Korea, China, and Thailand draw practice‐based recommendations and cross‐country perspectives on ultrasound‐guided filler injections of the upper face. Through the presented methodology and supplementary demonstration videos featuring a healthy volunteer and a cadaveric specimen, we present the anatomic landmarks useful for safe filler placement, which could prevent inadvertent intravascular injection of HA filler material and support efficient HA filler delivery in the intended facial layer.

## 2 Materials and Methods

### 2.1 Subjects

The volunteer in the proceeding report gave informed consent in advance of procedures to receive HA filler injections for temple hollowness, depressed forehead, and sunken eye under ultrasonographic guidance and agreed to the use and analysis of their data. Clinical injection techniques were conducted on one cadaveric specimen (a 72‐year‐old Korean male) to verify and evaluate applicable safety and efficacy. All procedures were conducted in compliance with the Declaration of Helsinki of the World Medical Association (WMA).

### 2.2 Ultrasound

A real‐time 2‐dimensional ultrasound device (Vscan Air, GE HealthCare, Chicago, Illinois, USA) with a B‐mode high‐frequency linear‐array transducer (12 MHz, width 40 mm) was used to view important structures in various planes in both the volunteer and the cadaveric specimen.

### 2.3 Filler

The fillers used in this study were Ministry of Food and Drug Safety (MFDS)‐approved HA fillers containing 20 mg/mL HA with 0.3% (3 mg/mL) lidocaine (YVOIRE Volume Plus, LG Chem, South Korea) and 20 mg/mL of HA with 0.3% (3 mg/mL) lidocaine (YVOIRE Classic Plus, LG Chem, South Korea).

### 2.4 Data Collection

Photographs were taken before and after the procedures using a mounted digital camera (Alpha 7S III, Sony, Japan). Videos were recorded using a handheld interchangeable lens digital camera and a professional camcorder (FX3 Cinema Line and HXR‐NX80, Sony, Japan). Concurrently, 3‐dimensional facial soft tissue was analyzed using stereophotogrammetric images (LifeViz Mini Face, QuantifiCare, France).

Anatomic landmarks, injection techniques, and safety guidelines were discussed alongside images and videos of the cadaveric specimen. The lead authors proposed statements on treatment goals and current practice in upper‐face HA‐filler use, based on their experience and with reference to peer‐reviewed literature, and received input from the global panel members.

### 2.5 Cadaveric Dissection

HA filler, premixed with green and blue dye (Davidson Tissue Dye, Bradley Products, Indiana, USA), was injected to visualize and differentiate the injection planes during the cadaveric specimen dissection. The injected areas were analyzed layer by layer to reveal the site of HA filler injection, deposited via superficial‐plane and deep‐plane techniques.

### 2.6 Expert Recommendations

The authors shared videos, anatomical diagrams, literature, and methodology at an online meeting where techniques for the upper facial regions were presented for group review and input. Further refinement and input were provided during the development of these recommendation statements via email and video conferencing.

## 3 Results and Discussion

### 3.1 Benefits of the Ultrasound‐Guided Filler Injection Technique

Solid structures (e.g., skin, adipose tissue) appear hyperechoic or white, while fluids (e.g., blood) appear anechoic or black on ultrasound. Vessels may appear linear or circular depending on the orientation of the transducer [9]. Upon injection, HA fillers first result in anechoic, poorly defined ultrasound images of globular distribution [14]. This changes as the filler integrates into the various layers of the skin [15, 16]. Real‐time visualization of the interaction between fillers and soft tissue provides the following benefits [9, 15]:

- Confirmation of the injection plane during and after filler placement
- Mapping of vessels before and after injection (reducing the risk of damage)
- Determination of anatomic variations in the vasculature (enhancing procedural safety)
- Prevention of post‐injection vessel compression and tension on the facial nerves

Apart from anatomical mapping, ultrasonographic reversal of dislocation and overcorrection may be performed, as well as treating vascular compromise with hyaluronidase [9].

Ultrasound usage requires an understanding of the technique's scientific principles [13] and the anatomy of the field examined, as well as practical skills such as operator dexterity and the ability to manipulate a tethered or hand‐held probe while injecting [15].

*Recommendation 1.* Ultrasonographic mapping of critical structures prior to and after filler injection may minimize the risk of adverse effects. The ultrasound device should have a frequency of at least 12 MHz as a minimum requirement.

### 3.2 Anatomy of the Upper Face

Age‐related changes in the skin and subcutaneous tissue of the upper face contribute to an older appearance that may be corrected with HA fillers [14, 17]. Older individuals tend to have age‐related elastolysis and collagen degradation manifesting as brow ptosis and deep forehead furrows [18]. Bony resorption and atrophy of the facial fat and muscles can lead to a flattening of facial angles, leading to a decrease in the prominence of the lower forehead and hollowing of the temples [19]. These changes contribute to an overall aged appearance [20].

The upper face is bounded superiorly by the hairline and inferiorly by the glabella, brow, and supraorbital ridge [17]. Laterally, the temporal line is a bony protuberance that provides the superior attachment area for the temporalis, often used to distinguish the central upper third (i.e., forehead) from the lateral upper part of the face (i.e., temporal area) [17]. The layers of tissue in the forehead are skin, subcutaneous tissue, muscles encased by the superficial musculoaponeurotic layer, a loose areolar connective tissue layer, and finally, the periosteum of the frontal bone [20]. The forehead muscles mainly comprise the frontalis, corrugator supercilii, depressor supercilii, procerus, and the orbital portion of the orbicularis oculi. The temporalis muscles are lateral to the frontalis [17].

The upper face is supplied by branches of the external and internal carotid arterial systems. Knowledge of these vascular structures and their common anatomical variations is of clinical significance when considering the risk of iatrogenic vascular thrombosis following soft tissue filler injections [17, 20, 21]. Further discussions relevant to the injection of fillers on specific landmarks and vascular structures in the forehead, temples, and upper section of the eyelids are presented in the following sections.

*Recommendation 2*. All injectors need to know and understand the gross anatomy of the upper face and the potential for anatomical variations in facial vasculature. This ensures the ability to tailor the technique according to the individual patients.

### 3.3 Anatomy‐Based Clinical Performances for Safety and Efficacy

#### 3.3.1 Forehead

##### 3.3.1.1 Landmarks and Treatment Indications

The supraorbital and supratrochlear arteries and their branches and the frontal branch of the superficial temporal artery that anastomoses with the supraorbital artery are the critical vascular structures in the forehead [20]. The supraorbital artery arises from the supraorbital foramen, located at the lower aspect of the orbital rim, running deep to the frontalis as the deep branch; it then penetrates the frontalis to run superficially as the superficial branch. Conversely, the supratrochlear artery emerges from the lower medial margin of the orbital rim and has superficial and deep branches that supply the superficial and deep aspects, respectively, of the frontalis near the midline of the forehead [7]. These arteries are situated from below the frontalis muscle until above the orbital rim (1.5–2.0 cm), where they pierce the muscle and become more superficial toward the hairline. Superiorly, the arteries lie in a subcutaneous plane; hence, fascia, fat, and the frontalis muscle protect the vessels if a cannula is inserted properly in the supraperiosteal plane of the upper forehead [20].

Ultrasonographic visualization of arteries in the forehead is essential to detect variations in layer thickness and the location of critical vessels such as the supraorbital artery and supratrochlear arteries as they emerge from their respective foramina and through the frontalis muscle.

##### 3.3.1.2 Technique

**FIGURE 1:**
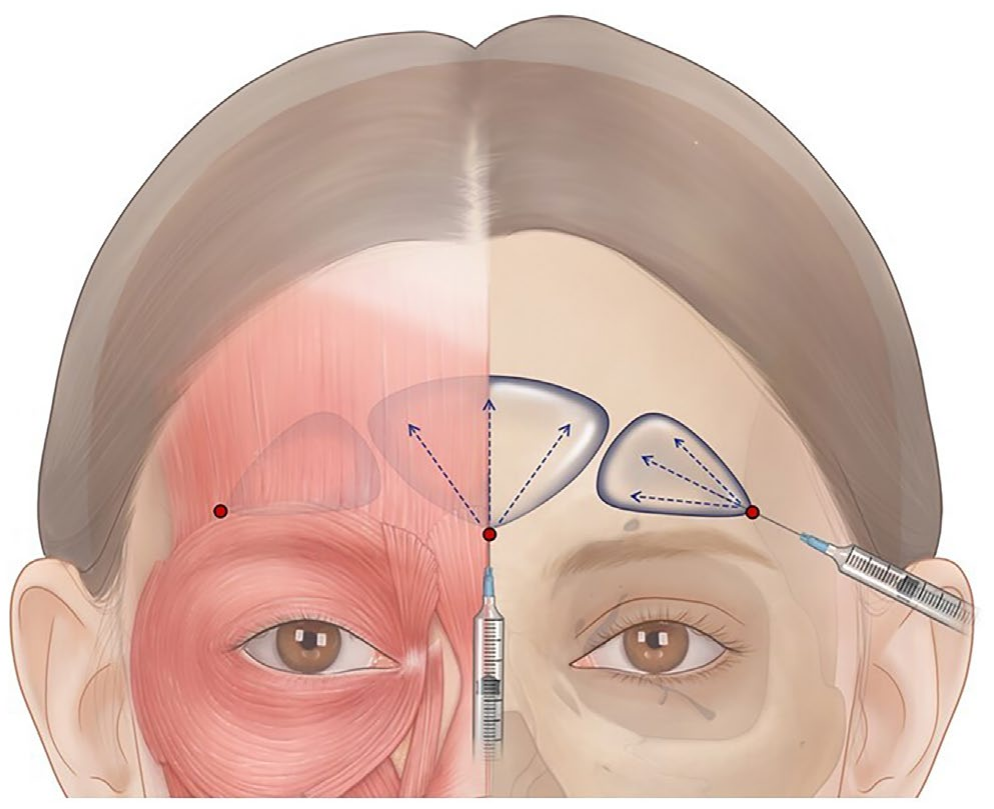
Injection technique for forehead filler treatment (entry points are represented by red dots).

To conduct a nerve block of the forehead, inject a local anesthetic at the needle entry points (Figure 1) and wait for 15–20 min, as the area may be sensitive to pain. Typically, fillers should be administered deep in the supraperiosteal plane using a blunt tip cannula (e.g., 25G or 23G, 5 cm). Deep introduction of the cannula is essential and is achieved by ensuring that the cannula touches the frontal bone [20]. Upon ensuring adequate distance of the cannula from the critical vessels in the correct layer, the probe may be removed and the filler injected.

To correct the upper part of the forehead, an additional entry point could be positioned at the hairline, approximating the region of the metopion (1*–*2 cm from the hairline) and at the deepest layer (i.e., supraperiosteal) to avoid the vessels in the subcutaneous layer [20]. As with non–ultrasound‐guided treatment, it is advised to aspirate at the first injection. It may be challenging to maintain the cannula in a single injection plane; hence, it is necessary to stabilize the cannula tip, maintaining it in the correct plane upon insertion [20]. After treatment, check the product location and ensure that there is no extravascular compression.

**FIGURE 2:**
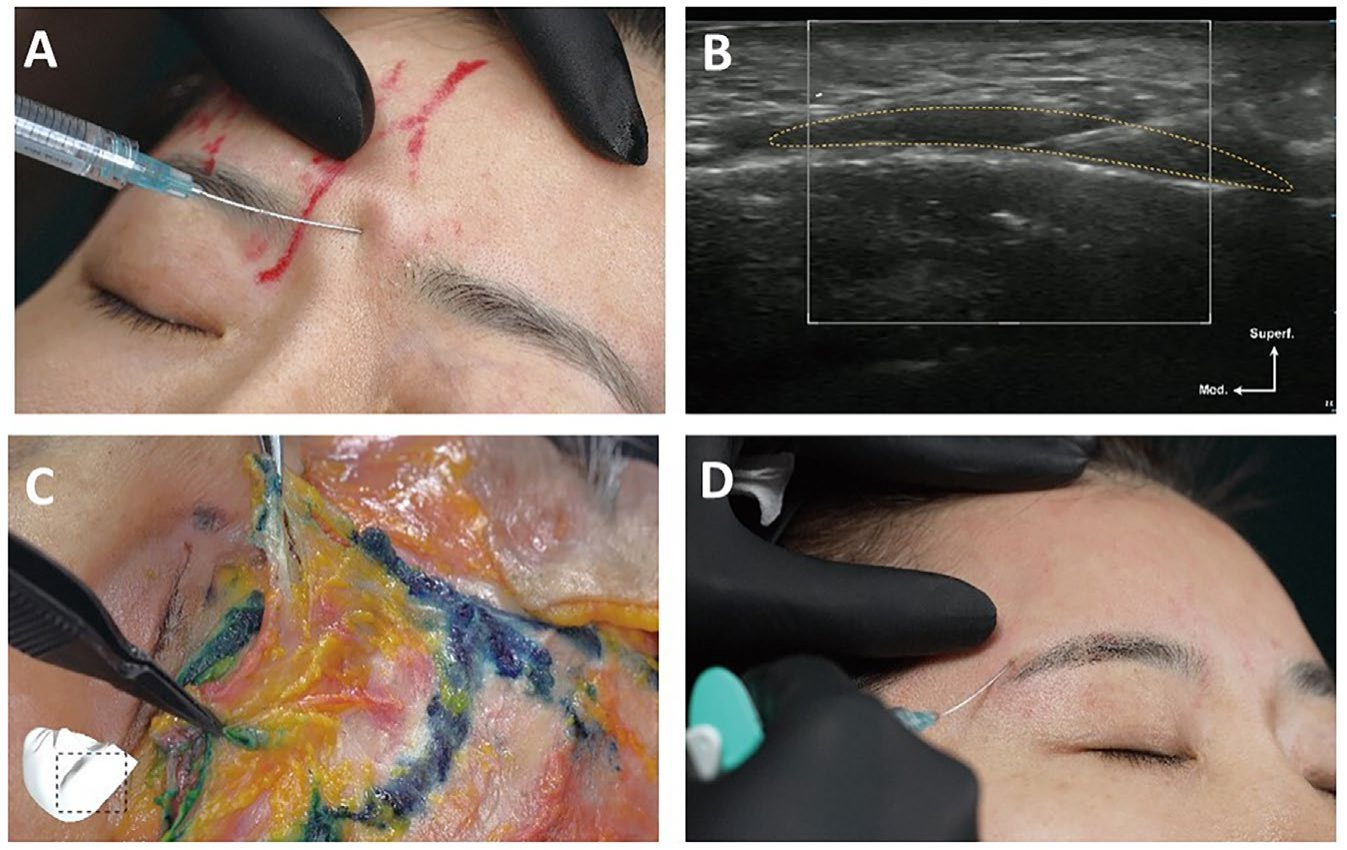
A demonstration of the midpoint approach for the forehead (41‐year‐old Korean female). (A) Clinical injection. (B) Clinical ultrasound. (C) Cadaver dissection (72‐year‐old Korean male). (D) A photo of the lateral approach for the forehead.

*Recommendation 3–1*. Guided by ultrasound mapping, a blunt tip cannula may be placed in the entry point either in the midline between the medial aspects of the brows or 1 cm from the lateral brow margins above the orbital rim at the area of the temporal crest (Figures 1, 2; Video S1), slowly delivering 2–5 mL of filler material (depending on the treatment area and patient's needs) using the fanning technique.

#### 3.3.2 Temple

##### 3.3.2.1 Landmarks and Treatment Indications

The superficial temporal fascia is supplied by the superficial temporal artery, which connects to the supraorbital artery [20]. The anterior deep temporal artery connects with the zygomaticotemporal artery, which anastomoses with the ophthalmic artery [20]. The medial zygomaticotemporal vein (MZTV) has a highly variable topography [22]; as it drains into the middle temporal vein, its location and thickness (up to 2 mm) make it particularly susceptible to vascular compromise during injections [22]. The temporal ridge/crest and zygomatic arch represent important landmarks around the temple; from these, a cannula can be directed between the deep and the superficial temporal fascia. Ultrasound can determine the vascular‐free regions in this area.

##### 3.3.2.2 Technique

**FIGURE 3:**
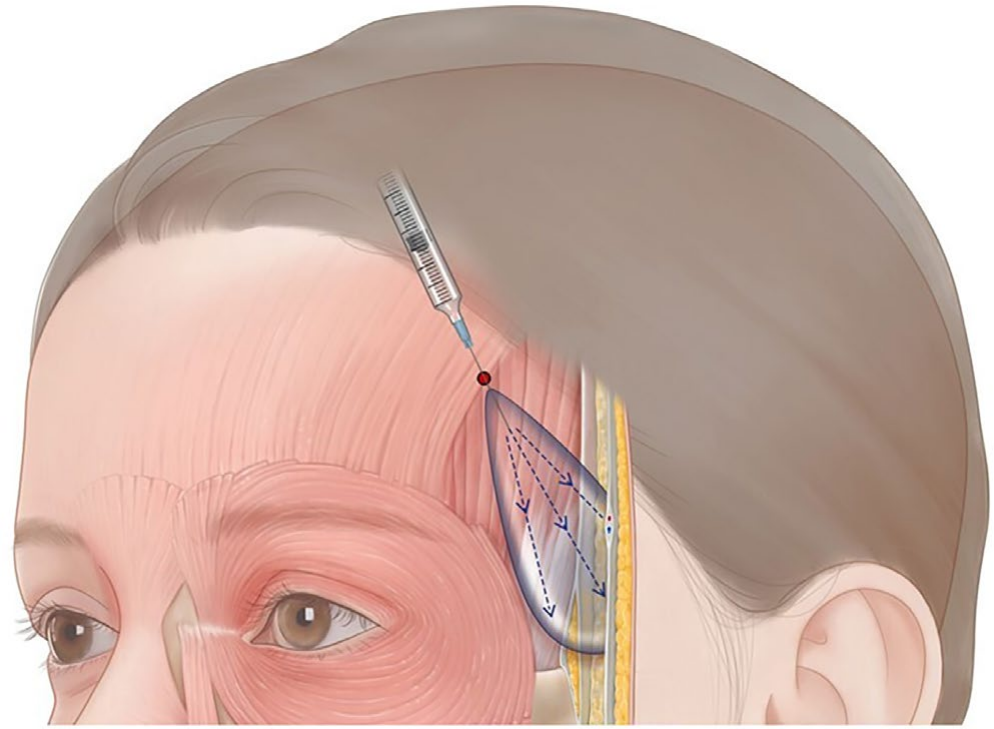
Injection technique for temple filler treatment. Superficial plane: Between superficial temporal fascia and deep temporal fascia.

Local anesthesia is injected at entry points for the needle or cannula injection (Figure 3), which are at the temporal ridge/crest and/or near the zygomatic arch. The superior area can be filled to lift the lateral brow. Lateral cheek lifting to enhance mandible and neck definition may be accomplished by injecting into the inferolateral area. The area between these two may be treated to improve anterior temple lifting by stretching the lower eyelid [20]. Subdermal or interfacial injection planes may be filled to correct posterior and anterior temple volume loss. Subdermal injections may correct posterior and anterior temple deficits with visible temporal blood vessels and very thin skin [20]. For severe anterior temple volume loss, a 30G needle with the bevel facing down may be used to administer 0.5–1.0 mL of filler material to the supraperiosteal layer via 0.1 mL microbolus injections [20].

**FIGURE 4:**
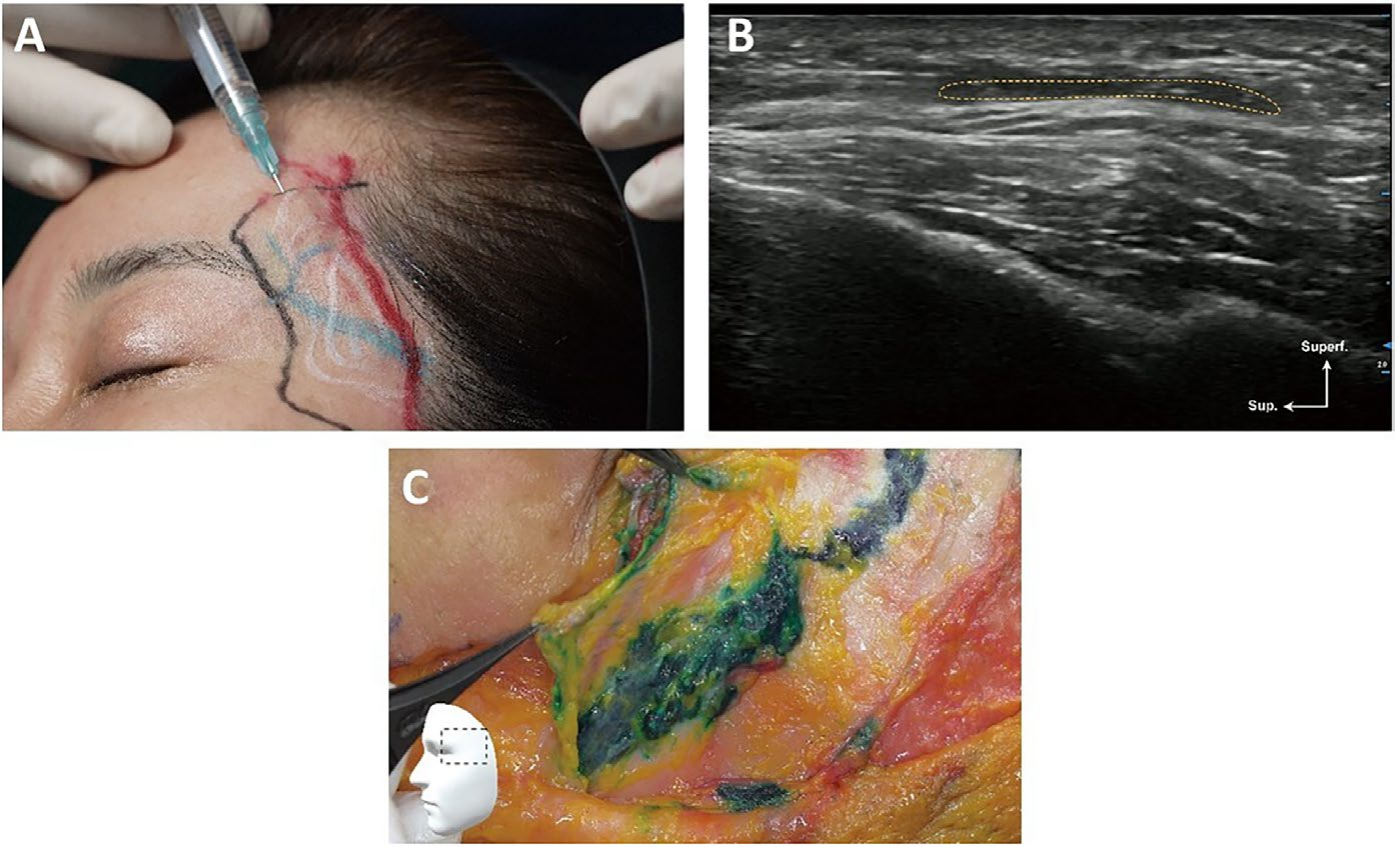
A demonstration of the superficial plane injection technique in the temple (41‐year‐old Korean female). (A) Clinical injection. (B) Clinical ultrasound. (C) Cadaver dissection (72‐year‐old Korean male).

A combination approach using both needle (e.g., 27G) and cannula (e.g., 22G) may be considered to supplement bone volume and improve the lifting effect and to smoothen the temporal contour, respectively [20]. Avoid entry‐point scarring by using multiple ports in several locations. Although more challenging than the temporal‐crest approach, the inferior approach from the zygomatic arch with a longer cannula and ultrasound guidance allows injectors to reach the upper temporal space and extend into the retro‐orbicularis oculi fat for additional brow lift and the lateral orbital rim contouring, as necessary [4].

The safety of prior syringe aspiration before injecting in the temple is contentious [20]; however, this technique, along with ultrasonographic evaluation of the temporal vessels, may be useful to avoid directly damaging these critical structures.

After treatment, surface irregularities may arise. A gentle massage of the injected area can correct these [4], and adjustment of position and filler volume in multiple layers may lessen such irregularities. Some patients may experience mild, transient headaches [23]. In rare instances, headaches may signal vascular compromise [24].

**FIGURE 5:**
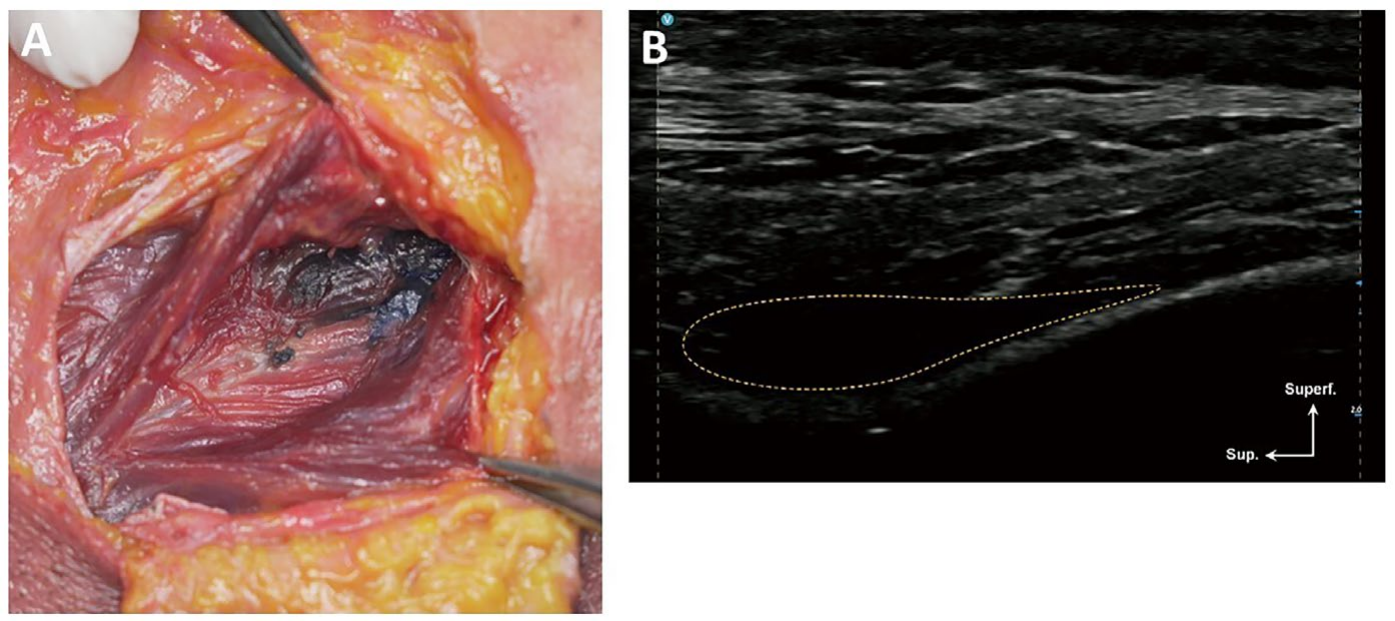
A demonstration of filler placement following the deep plane injection. technique in the temple (72‐year‐old Korean male). (A) Cadaver dissection. (B) Cadaver ultrasound.

*Recommendation 3–2*. Using ultrasound, it is possible to view the anterior and posterior deep temporal arteries, as well as the deep and superficial temporal fasciae. Through slow injection of filler through either the temporal crest or the zygomatic arch insertion points using a blunt cannula (e.g., 22G or 26G) via the fanning technique, about 1–2 mL of filler material may be placed in one side (Figures 3, 4, 5; Video S2) either through the superficial‐plane or the deep‐plane injection approach. Ultrasound can be used to check filler positioning in the temple region, reverse unwanted outcomes, and improve patient satisfaction.

#### 3.3.3 Upper Eyelids (Sunken Eyes)

##### 3.3.3.1 Landmarks and Treatment Indications

The most important vessels in the upper face supplying the upper eyelid include the supraorbital artery (which emerges through the supraorbital notch or foramen), the supratrochlear artery (which exits from the frontal notch at the medial aspect of the superior orbital rim, supplying the forehead), and the more medial angular and dorsal nasal arteries. These arise from the ophthalmic artery as it emerges from the orbital septum inferior to the medial aspect of the superior orbital rim [20].

Ultrasonographic guidance is used for determining the preinjection cannula depth and the adequate placement and amount of filler in the appropriate layer. In addition, ultrasonographic evaluation of the medial part of the upper lid enables identification of the ophthalmic artery emerging points and the location of the orbital septum to avoid injuring these structures [25].

##### 3.3.3.2 Technique

**FIGURE 6:**
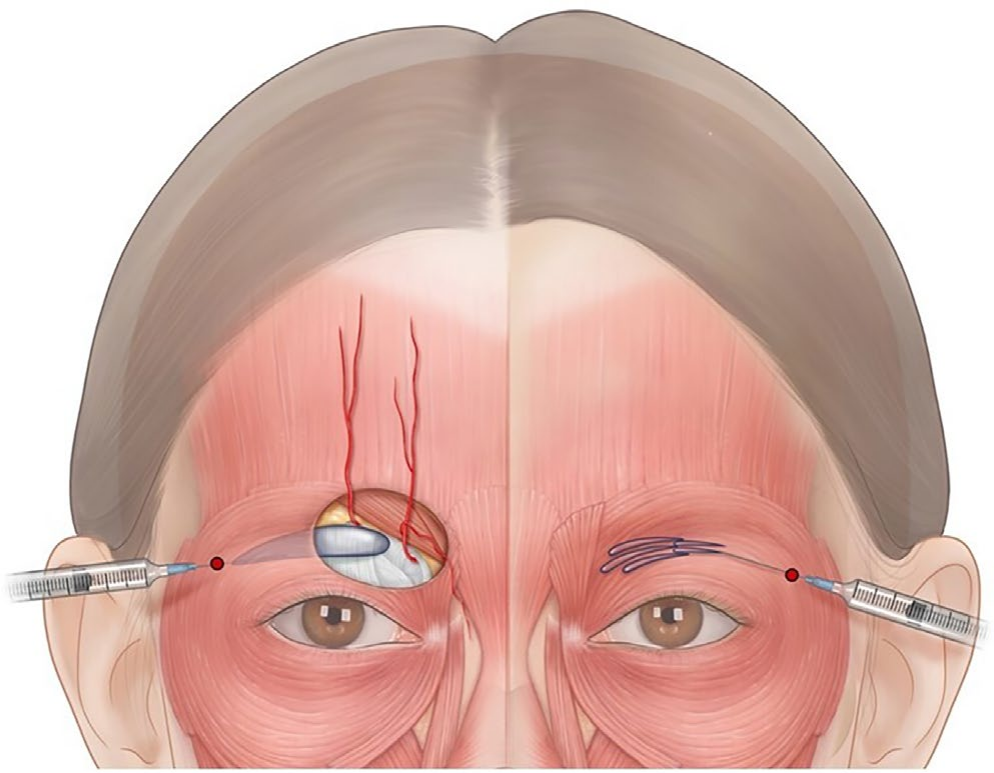
Injection technique for filler treatment of sunken eyes.

Although sharp needles offer the advantage of precise placement and require less technical expertise [15], we suggest using a 25G–23G blunt cannula because of its relative safety compared with the technique employing sharp needles (Figure 6). However, an improperly positioned straight cannula may curve outward and perforate the muscle and dermis while being inserted medially from a lateral entry point. Subcutaneous injection of fillers in this area may introduce lumps (i.e., more apparent with eyes open), thick upper eyelids, and even eyelid ptosis. Hence, it is essential to practice care when performing the technique [12] and use smaller volumes (e.g., ≤ 0.5 mL) of low G‐prime and non–cross‐linking HA fillers.

It is important to prevent the cannula from coming into contact with the supraorbital notch, or foramen, because of the proximity of the supraorbital artery to the bone [12].

**FIGURE 7:**
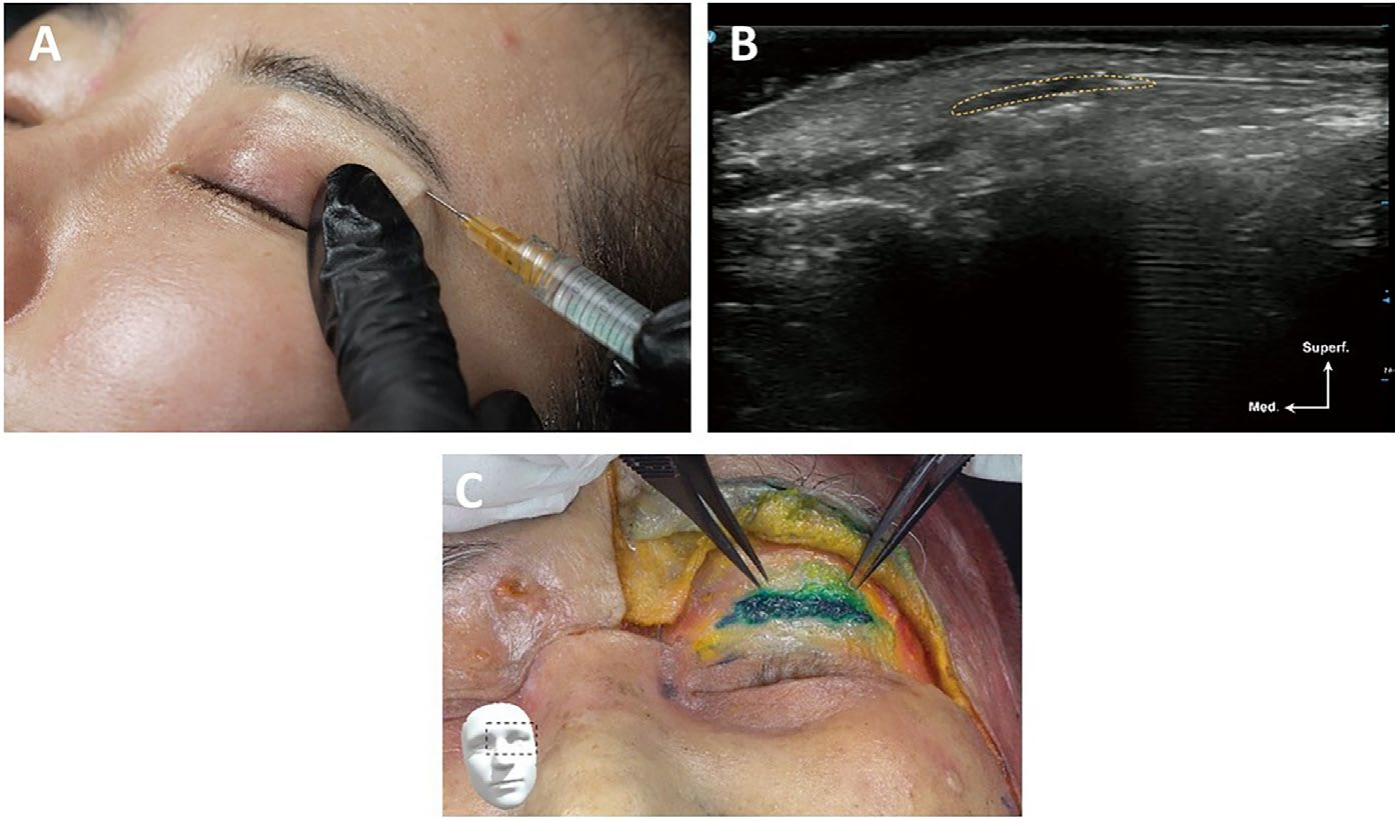
A demonstration of superficial plane injection of filler in the upper eyelid (41‐year‐old Korean female). (A) Clinical injection. (B) Clinical ultrasound. (C) Cadaver dissection (72‐year‐old Korean male).

**FIGURE 8:**
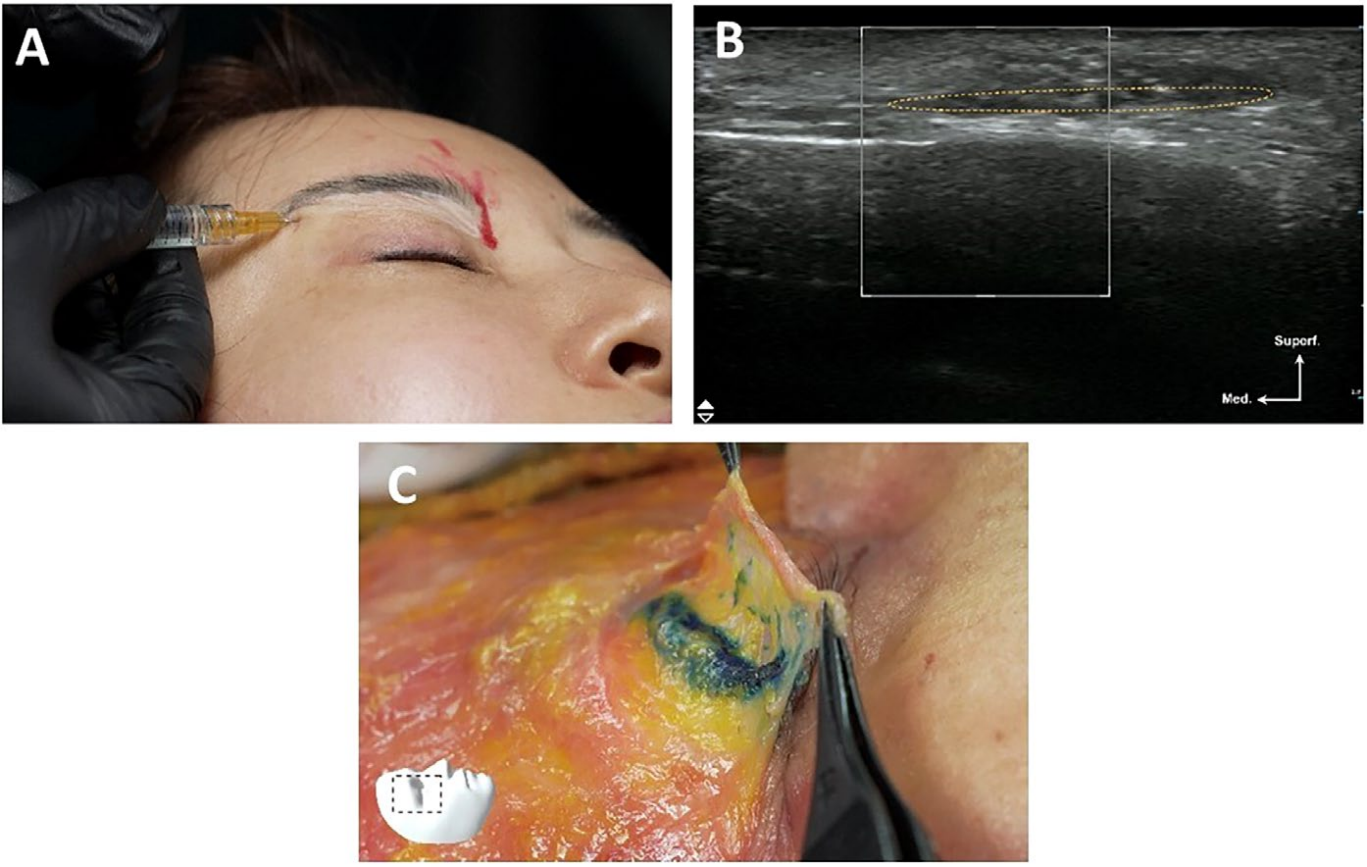
A demonstration of deep‐plane injection of filler in the upper eyelid (41‐year‐old Korean female). (A) Clinical injection. (B) Clinical ultrasound (C) Cadaver dissection (72‐year‐old Korean male).

*Recommendation 3–3*. Ultrasound may be used to avoid critical vessels when advancing the cannula from under the lateral brow to the medial area to prevent hematoma and/or other bleeding complications when correcting sunken eyes. The needle entry point is identified at the crossing of the vertical line traced from the lateral canthus to the orbital rim (Figure 7). Optional topical anesthesia is administered, followed by local 2% lidocaine (with 1:100000 epinephrine) per side. The patient is positioned in a semireclining position during the procedure (Video S3). Using a 27G–25G cannula, a maximum of 0.5 mL soft filler material is administered per side, mainly into the submuscular or preseptal plane (i.e., deep plane injections at the retro‐orbicularis oculi fat) (Figure 8) via slow retrograde linear injections; if needed, small volumes (e.g., ≤ 0.1 mL) of low G‐prime filler may be placed at the subdermal plane (superficial plane injections) using a 30G–27G cannula to smoothen the surface (Figure 7). Gentle molding using a standard ointment can smoothen the skin surface.

### 3.4 Additional Recommendations

*Recommendation 4*. Pockets of HA fillers can be visualized with ultrasound. If needed, hyaluronidase can be directly injected via ultrasound‐guided delivery into the filler pocket that is causing the deformity [9].

### 3.5 Limitations

This study is limited to practice‐based recommendations and existing literature. No studies have been published evaluating the long‐term effects and safety of ultrasound‐guided filler administration. More information regarding technical specifications and the long‐term effects of ultrasound‐guided methods may be needed. Guidelines on ultrasound‐guided filler placement into other anatomic locations (e.g., tear trough, jawline, nasolabial folds) may be forthcoming.

## 4 Conclusions

Detailed knowledge of the anatomy of the forehead, temple, and periorbital area is essential in filler administration. In cases where anatomic variations exist, the use of ultrasound as a guide for filler placement provides an additional safety measure for injectors. Adverse events may be reversed through ultrasonographic‐guided treatment (i.e., using hyaluronidase). Although ultrasound‐guided filler placement is still in its infancy and the techniques outlined in this paper are limited to a few cases, current evidence appears promising and may give rise to standard practices incorporating ultrasound in protocols for preventive and therapeutic management of adverse events.

## Author Contributions

All authors participated in the study design, research, analysis, and drafting of the manuscript, and gave approval for the study.

## Ethics Statement

The authors have nothing to report.

## Consent

Consent has been given by the patient who appears in the photographs/videos associated with this manuscript.

## Conflicts of Interest

Beyond this study, J.Y.J., H.C., S.C.H., R.W., Q.L., L.Z., and H‐.J.K. declare no conflicts of interest. J.I.N. and W.E.J. are full‐time employees of LG Chem Co. Ltd.

## Supporting information


Video S1.

Video S2.

Video S3.

## Data Availability

The data that support the findings of this study are available on request from the corresponding author. The data are not publicly available due to privacy or ethical restrictions.
